# Clinical Characteristics of Psoriasis Cases Treated by Biologics With an Extended Administration Interval: A Single-Center, Retrospective Observational Study

**DOI:** 10.7759/cureus.59969

**Published:** 2024-05-09

**Authors:** Hiroyoshi Nozaki, Masaru Honma, Akemi Ishida-Yamamoto

**Affiliations:** 1 Dermatology, Asahikawa Medical University, Asahikawa, JPN; 2 International Medical Support Center, Asahikawa Medical University, Asahikawa, JPN

**Keywords:** psoriasis, interleukins, interval, body mass index, biologic

## Abstract

Background

Psoriasis is a chronic inflammatory skin disease with multiple organ manifestations such as arthritis and cardiovascular diseases. While recent therapeutic advancements in systemic biologics have demonstrated efficacy against psoriasis, a complete cure has not been achieved and patients require lifelong treatment to control symptoms.

Objective

This study aimed to clarify the clinical characteristics of psoriasis patients treated with biologics at an extended interval.

Methods

This study included patients with psoriasis who were administered biologic therapy for longer than the standard interval (at least a week) and who objectively maintained favorable conditions (static Physician’s Global Assessment ≤ 0 to 1). Clinical characteristics, such as body weight (BW), body mass index (BMI), and body fat percentage, were compared to those of patients who were administered biologic therapy at standard intervals.

Results

Among 162 Japanese patients with psoriasis, 35 were treated with biologics at extended intervals. In the group with extended treatment intervals, patients treated with interleukin (IL)-17 inhibitors (n = 15) presented statistically lower BMI than those treated with IL-23 inhibitors (n = 17) (*P* < 0.016). The group treated with IL-17 inhibitors at extended intervals showed significantly lower BMI and body fat percentage than the group at standard intervals (*P* < 0.05).

Conclusion

Trends in our hospital suggest that psoriasis patients with low BMI and body fat percentage can maintain good status with extended interleukin (IL)-17 inhibitor dosing intervals (static Physician’s Global Assessment ≤ 0 to 1).

## Introduction

Psoriasis is a chronic inflammatory disease that presents with scaly reddish plaques on repeatedly chafed body parts [1]. Additionally, it presents with other multiorgan comorbidities such as arthritis and cardiovascular diseases. Recently, systemic involvement in patients with psoriasis has been recognized as “psoriatic disease”, which includes uveitis, inflammatory bowel diseases, cardiac disorders, and metabolic syndrome [2].

Recent progress in systemic therapeutic options for psoriasis has allowed successful treatment of skin lesions and prevention of progression of comorbidities related to psoriasis [3]. However, a cure for psoriasis has not been achieved, and patients require appropriate treatment to control their symptoms throughout life. Considering the economic, physical, and psychological burden on these patients, identifying clinical markers reflecting reduced therapeutic efficacy is important. While the production of anti-drug antibodies should be considered [4], extending the administration intervals in biologic therapy is one of the options in clinical practice.

This study aimed to clarify the clinical characteristics related to an extended interval of biologic therapy in patients with psoriasis.

## Materials and methods

Patients with psoriasis treated with biologics

This study is real-world and retrospective and includes psoriasis patients who started treatment with biologic agents at Asahikawa Medical University Hospital between January 2010 and December 2021 and were continuing treatment as of December 2021. The patients were allocated to one of two groups: one treated at standard intervals and the other treated at extended intervals. The extended interval group included patients with psoriasis who were treated with biologics at an interval longer than the standard (regularly extended by at least one week, excluding accidental extensions) than the standard treatment interval at the patient's request and objectively maintained favorable conditions (static Physician’s Global Assessment ≤ 0 to 1) as of December 2021 [5,6]. Many of the patients who had a poor prognosis (static Physician’s Global Assessment > 2) after the extended dosing interval were assigned to either the extended or standard group because they were able to maintain favorable conditions or return to the standard dosing interval by shortening the extended interval. The study was performed in accordance with the Declaration of Helsinki and Good Clinical Practice guidelines and approved by the ethics committee of Asahikawa Medical University.

Clinical information

Clinical information, including age, sex, subtypes of psoriasis, Psoriasis Area and Severity Index (PASI) score, treatment conditions, body weight (BW), body mass index (BMI), and body fat percentage, were retrospectively collected from electronic medical records.

Statistical analysis

Statistical analysis was performed using EZR (Saitama Medical Center, Jichi Medical University, Saitama, Japan) [7]. To compare independent groups, the Mann-Whitney U test and Fisher’s exact tests (statistical significance, P < 0.05) or the Bonferroni correction (statistical significance, P < 0.016) were employed.

## Results

**Figure 1:**
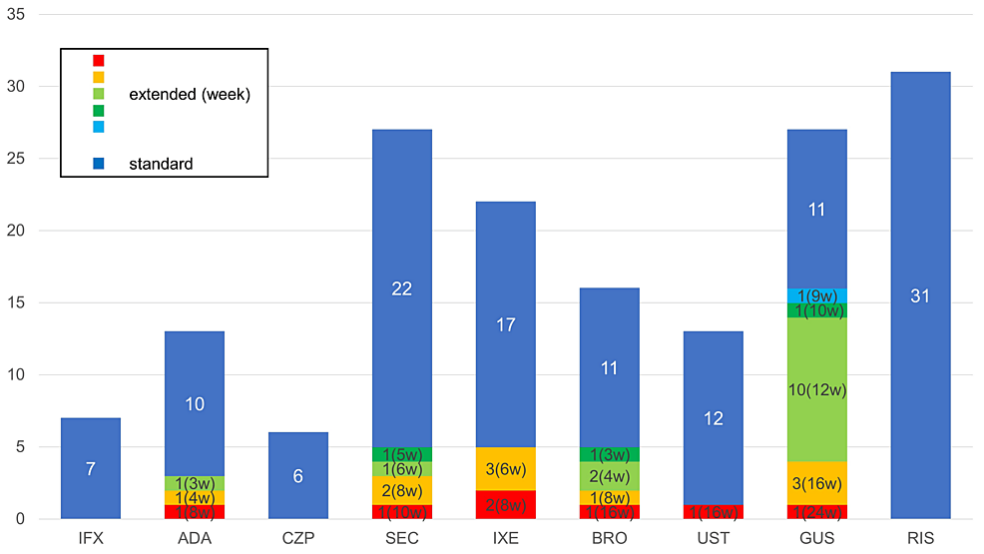
Breakdown of biologics used in patients with psoriasis. Figure 1. Breakdown of biologics used in patients with psoriasis. IFX, infliximab; ADA, adalimumab; CZP, certolizumab pegol; SEC, secukinumab; IXE, ixekizumab; BRO, brodalumab; UST, ustekinumab; GUS, guselkumab; RIS, risankizumab.

In this study, 162 Japanese patients with psoriasis (42 females and 120 males; median age, 57 years) were included, and the proportions of psoriasis subtypes were 80.2%, 9.3%, 3.1%, and 34.6% for psoriasis vulgaris, generalized pustular psoriasis, erythrodermic psoriasis, and psoriatic arthritis, respectively. In 35 patients, biologics were administered at extended intervals (5 females and 30 males; median age, 58 years), and the proportions of psoriasis subtypes were 82.9%, 2.9%, 0%, and 28.6% for psoriasis vulgaris, generalized pustular psoriasis, erythrodermic psoriasis, and psoriatic arthritis, respectively. The biologics used for treatment were tumor necrosis factor (TNF)-α inhibitor in three cases (3 of 13 adalimumab cases, none of 7 infliximab cases, and none of 6 certolizumab pegol cases), interleukin (IL)-17 inhibitors in 14 cases (5 of 27 secukinumab cases, 5 of 22 ixekizumab cases, 5 of 16 brodalumab cases), IL-12/IL-23 p40 inhibitor in one case (1 of 13 ustekinumab cases), and IL-23 p19 inhibitor in 16 cases (16 of 27 guselkumab cases and none of 21 risankizumab cases) (Figure 1).

**Figure 2:**
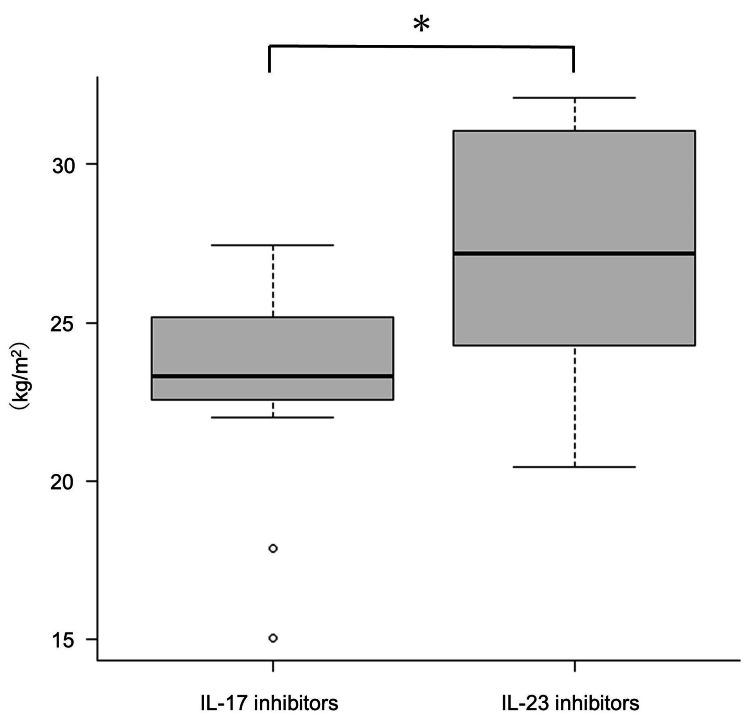
Among patients with psoriasis treated with extended drug intervals, those treated with IL-17 inhibitors show significantly lower BMI than those treated with IL-23 inhibitors Among patients with psoriasis treated with extended drug intervals, those treated with IL-17 inhibitors show significantly lower BMI than those treated with IL-23 inhibitors. * Significant difference with p-value < 0.016. IL, interleukin; BW, body weight; BMI, body mass index

There were no significant differences in BW (n = 153), BMI (n = 147), and body fat percentage (n = 104) between the groups treated at standard and extended intervals (P = 0.93, 0.24, and 0.14, respectively). Among the cases with extended administration intervals, BMI (median, 23.3 kg/m2) was significantly lower in patients treated with IL-17 inhibitors than those treated with IL-23 p19 or p40 inhibitors (median, 27.2 kg/m2; P = 0.011) but not BW or body fat percentage (P = 0.023 and 0.12, respectively) (Figure 2). The BW, BMI, and body fat percentage of the standard interval treatment group of IL-17 inhibitors did not differ from those of IL-23 p19 or p40 (P = 0.37, 0.85, and 0.37, respectively).

**Figure 3:**
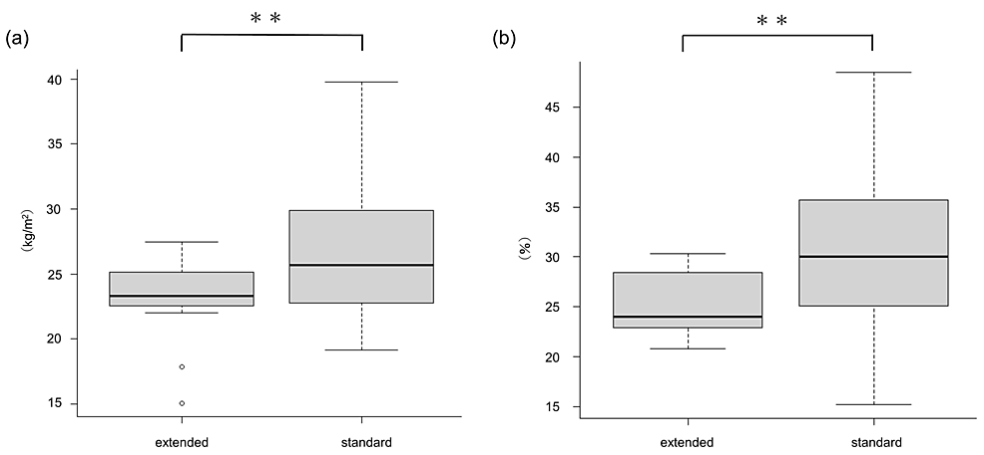
Patients with psoriasis in the extended interval groups treated with IL-17 inhibitors show significantly lower (a) BMI and (b) body fat percentage than patients in the respective standard interval groups Patients with psoriasis in the extended interval groups treated with IL-17 inhibitors show significantly lower (a) BMI and (b) body fat percentage than patients in the respective standard interval groups. ** Significant difference with p-value < 0.05. IL, interleukin; BMI, body mass index

Among IL-17 inhibitor-treated cases, BMI (median, 23.3 kg/m^2^) and body fat percentages (median, 24.0%) were significantly lower in the extended interval group than in the standard interval group (median, 25.7 kg/m^2^ and 30.0%; P = 0.039 and 0.031, respectively) but not BW (P = 0.13) (Figure 3). There were no significant differences in BW, BMI, or body fat percentage among the secukinumab extended intervals, ixekizumab extended intervals, and brodalumab extended intervals groups in IL-17 inhibitor-treated cases (data not shown).

We have shown the result of the consideration in various combinations (Table 1).

**Table 1:** Statistical analysis (median and P-value) of the effect of treatment intervals and type of biologic therapy on BW, BMI, and body fat percentage of participants with psoriasis TNF, tumor necrosis factor; IL, interleukin; BW, body weight; BMI, body mass index Significant difference with * P-value < 0.016 (Bonferroni correction); ** P-value < 0.05

	BW (kg)		BMI (kg/m^2^)		body fat percentage (%)	
	median (min-max)	P-value	median (min-max)	P-value	median (min-max)	P-value
all (extended)	69.0 (39.0-100.0) (n=33)	0.93	24.7 (15.0-32.1) (n=32)	0.24	25.3 (16.8-38.1) (n=22)	0.14
vs all (standard)	68.4 (38.0-157.0) (n=120)		25.3 (15.2-67.1) (n=115)		27.6 (0.3-65.7) (n=82)	
TNF inhibitors (extended)	60.0 (54.0-76.6) (n=3)	0.62	21.1 (20.8-25.9) (n=3)	0.31	24.7 (23.9-25.5) (n=2)	0.69
vs TNF inhibitors (standard)	64.5 (45.7-120.0) (n=22)		24.2 (18.9-51.9) (n=21)		26.2(18.8-63.1) (n=13)	
IL-17 inhibitors (extended)	68.0 (39.0-82.7) (n=15)	0.13	23.3 (15.0-27.4) (n=15)	0.039**	24.0 (20.8-30.3) (n=10)	0.031**
vs IL-17 inhibitors (standard)	69.5 (45.5-115.0) (n=44)		25.7 (19.2-39.8) (n=43)		30.0 (15.2-48.5) (n=33)	
IL-23 inhibitors (extended)	75.0 (53.0-100.0) (n=15)	0.093	27.2 (20.4-32.1) (n=14)	0.36	26.7 (16.8-38.1) (n=10)	0.93
vs IL-23 inhibitors (standard)	68.9 (38.0-157.0) (n=54)		25.7 (15.2-67.1) (n=51)		27.1 (0.3-65.7) (n=36)	
TNF inhibitors (extended)	60.0 (54.0-76.6)	1.00	21.1 (20.8-25.9)	0.50	24.7 (23.9-25.5)	0.76
vs IL-17 inhibitors (extended)	68.0 (39.0-82.7)		23.3 (15.0-27.4)		24.0 (20.8-30.3)	
TNF inhibitors (standard)	64.5 (45.7-120.0)	0.24	24.2 (18.9-51.9)	0.57	26.2(18.8-63.1)	0.53
vs IL-17 inhibitors (standard)	69.5 (45.5-115.0)		25.7 (19.2-39.8)		30.0 (15.2-48.5)	
TNF inhibitors (extended)	60.0 (54.0-76.6)	0.19	21.1 (20.8-25.9)	0.12	24.7 (23.9-25.5)	0.27
vs IL-23 inhibitors (extended)	75.0 (53.0-100.0)		27.2 (20.4-32.1)		26.8 (16.8-38.1)	
TNF inhibitors (standard)	64.5 (45.7-120.0)	0.70	24.2 (18.9-51.9)	0.62	26.2(18.8-63.1)	0.73
vs IL-23 inhibitors (standard)	68.9 (38.0-157.0)		25.7 (15.2-67.1)		27.1 (0.3-65.7)	
IL-17 inhibitors (extended)	68.0 (39.0-82.7)	0.023	23.3 (15.0-27.4)	0.011*	24.0 (20.8-30.3)	0.12
vs IL-23 inhibitors (extended)	75.0 (53.0-100.0)		27.2 (20.4-32.1)		26.8 (16.8-38.1)	
IL-17 inhibitors (standard)	69.5 (45.5-115.0)	0.37	25.7 (19.2-39.8)	0.85	30.0 (15.2-48.5)	0.37
vs IL-23 inhibitors (standard)	68.9 (38.0-157.0)		25.7 (15.2-67.1)		27.1 (0.3-65.7)	

The rate of extended intervals was not gender specific (male 25.0% vs female 11.9%, P = 0.08) and did not differ between the groups of patients under 65 years old and those aged 65 years or older (22.2% vs 20.4%, P = 0.84). There was no significant difference in the proportion of extension in biologic-naïve patients from biologic-switched patients (27.2% vs 16.0%, P = 0.13). In addition, PASI scores prior to initiation of the first biologic in the extended interval group were similar to those in the standard group (median, 13.9 vs 12.9, P = 0.18) (Table 2).

**Table 2:** Statistical analysis of the effect of treatment interval on sex, age, number of biologics experienced, and pre-treatment PASI score of psoriasis patients (percentage, median, and P value) PASI: Psoriasis Area and Severity Index

		extended	standard	P-value
sex	male (%)	25.0	75.0	0.085
	female (%)	11.9	88.1	
age (years)	< 65 (%)	22.2	77.8	0.84
	≧ 65 (%)	20.4	79.6	
biologics	naïve (%)	27.2	72.8	0.13
	switch (%)	16.0	84.0	
PASI (median)		13.9	12.9	0.18

## Discussion

Recent progress in the therapeutic options for psoriasis has allowed the successful treatment of skin lesions; however, patients’ needs have not yet been fully addressed. One unresolved issue is the optimization of therapeutic strength. In this study, we revealed that lower BMI and body fat deposition could be clinical markers in favor of extending administration intervals in patients treated with IL-17 inhibitors.

Elevated BMI and high-fat deposition are known risk factors for psoriasis, and insufficient efficacy of systemic treatment, including biologic therapy [8,9]. These body composition scores can be candidate predictors of superior therapeutic response by extending treatment intervals. In this study, significantly lower BMI and body fat deposition were observed in the extended dose interval group treated with IL-17 inhibitors than in the standard dose interval group. The results of this study show that the patients in the extended interval group with lower BMI treated with IL-17 inhibitors can be maintained under favorable conditions (static Physician’s Global Assessment ≤ 0 to 1). Furthermore, among biologic-treated patients at extended intervals, patients treated with IL-17 inhibitors presented with significantly lower BMI than IL-23 inhibitor-treated patients. These results suggest that intervals in the group using IL-23 inhibitors, particularly guselkumab, were extended irrespective of the BW and BMI. The standard administration interval of guselkumab is at a maintenance phase of eight weeks; however, the interval is frequently extended to the same 12-week interval as other IL-23 inhibitors for economic reasons (Figure 1). Collectively, lower BMI and body fat deposition can reflect superior treatment results, which enables the extension of therapeutic intervals in patients with psoriasis treated with biologics.

As mentioned above, obesity and excess fat deposition can negatively affect the molecular pathogenesis of psoriasis. Excess white adipose tissue can release pro-inflammatory adipokines and various cytokines, which exacerbate Th17-mediated inflammatory reactions [10]. The high-fat and simple sugar-containing western-style diet also strengthens IL-17-related inflammatory responses [11]. The dysregulated expression of proinflammatory adipokines (TNF, IL-6, leptin, and chemerin) and anti-inflammatory adipokines (adiponectin and omentin) form the molecular basis of psoriasis. A European population-based cohort study revealed that a BMI of 27 kg/m^2^ or more possibly increases the risk of psoriasis, and the risk can increase to more than 10% when BMI increases to 28 kg/m^2^ [12]. The group that achieved minimum disease activity (MDA) of psoriasis using biologic therapy presented with a lower BMI (26.47 kg/m^2^) than the failed MDA group (BMI 28.53 kg/m^2^) [13]. Therefore, the approximately 2 kg/m2 difference in BMI observed in this study reflects reduced disease activity in biologic-treated cases.

There are several limitations in our study. The same definition is used for drugs with different dosing intervals. The administration intervals were elongated according to the patient’s request without randomization, and the cases without regular evaluation of static Physician’s Global Assessment were excluded from our study. Confounding factors other than BW, BMI, and fat percentage were not included in the analysis. Since this is a single-center study, this trend may not be general.

## Conclusions

In biologic therapy for psoriasis, extended dosing intervals should be considered to optimize therapeutic efficacy; clinicians may benefit from the fact that extended dosing intervals for IL-17 inhibitors are associated with lower BMI and body fat composition but not for IL-23 inhibitors. Elevated BMI and high-fat deposition are known risk factors for psoriasis, which may result in reduced efficacy of systemic treatment, including biologic therapy. Trends at our institution suggest that psoriasis patients who can maintain good status with extended dosing intervals, especially for IL-17 inhibitors, are those with lower BMI and body fat percentage.
